# Supplementary material for: Mapping the evolution of fertility support policies in China: A content and instrumental analysis
Source: PLoS One. 2025 Oct 9;20(10):e0332137. doi: 10.1371/journal.pone.0332137 (PMC12510515; doi:10.1371/journal.pone.0332137)
Supplement: S1 Appendix — (ZIP) [file pone.0332137.s001.zip › S1 Appendix. 226 original policy documents/152-中华人民共和国妇女权益保障法(FBM-CLI-1-5673).doc]

中华人民共和国妇女权益保障法

**发布部门：** [全国人民代表大会](https://www.pkulaw.com/law/chl?Aggs.IssueDepartment=101)

**发文字号：**中华人民共和国主席令第58号

**发布日期：**1992.04.03

**实施日期：**1992.10.01

**时效性：** [已被修改](https://www.pkulaw.com/law/chl?Aggs.TimelinessDic=03)

**效力级别：** [法律](https://www.pkulaw.com/law/chl?Aggs.EffectivenessDic=XA0101)

**法规类别：** [老少妇幼残保护](https://www.pkulaw.com/law/chl?Aggs.Category=01604)

**修改依据：**[全国人民代表大会常务委员会关于修改《中华人民共和国妇女权益保障法》的决定(2005)](javascript:TMP(59777,0,'chl'))

[2005-2018编注](https://www.pkulaw.com/history/compare/编注-122.html) [1992-2005编注](https://www.pkulaw.com/history/compare/编注-121.html)

中华人民共和国主席令
（第五十八号）

　　《中华人民共和国妇女权益保障法》已由中华人民共和国第七届全国人民代表大会第五次会议于1992年4月3日通过，现予公布，自1992年10月1日起施行。

中华人民共和国主席　杨尚昆
1992年4月3日

中华人民共和国妇女权益保障法
（１９９２年４月３日第七届全国人民代表大会第五次会议通过）

第一章　总则

　　**第一条**　为了保障妇女的合法权益，促进男女平等，充分发挥妇女在社会主义现代化建设中的作用，根据[宪法](https://www.pkulaw.com/chl/5c498812eb87061ebdfb.html#class=alink)和我国的实际情况，制定本法。

　　**第二条**　妇女在政治的、经济的、文化的、社会的和家庭的生活等方面享有与男子平等的权利。

　　国家保护妇女依法享有的特殊权益，逐步完善对妇女的社会保障制度。

　　禁止歧视、虐待、残害妇女。

　　**第三条**　保障妇女合法权益是全社会的共同责任。国家机关、社会团体、企业事业单位、城乡基层群众性自治组织，应当依照本法和有关法律的规定，保障妇女的权益。

　　国家采取有效措施，为妇女依法行使权利提供必要的条件。

　　**第四条**　国务院和省、自治区、直辖市人民政府，采取组织措施，协调有关部门做好妇女权益的保障工作。具体机构由国务院和省、自治区、直辖市人民政府规定。

　　**第五条**　中华全国妇女联合会和各级妇女联合会代表和维护各族各界妇女的利益，做好保障妇女权益的工作。

　　工会、共产主义青年团，应当在各自的工作范围内，做好保障妇女权益的工作。

　　**第六条**　国家鼓励妇女自尊、自信、自立、自强，运用法律维护自身合法权益。

　　妇女应当遵守国家法律，尊重社会公德，履行法律所规定的义务。

　　**第七条**　对保障妇女合法权益成绩显著的组织和个人，各级人民政府和有关部门给予表彰和奖励。

第二章　政治权利

　　**第八条**　国家保障妇女享有与男子平等的政治权利。

　　**第九条**　妇女有权通过各种途径和形式，管理国家事务，管理经济和文化事业，管理社会事务。

　　**第十条**　妇女享有与男子平等的选举权和被选举权。

　　全国人民代表大会和地方各级人民代表大会的代表中，应当有适当数量的妇女代表，并逐步提高妇女代表的比例。

　　**第十一条**　国家积极培养和选拔女干部。

　　国家机关、社会团体、企业事业单位在任用干部时必须坚持男女平等的原则，重视培养、选拔女干部担任领导成员。

　　国家重视培养和选拔少数民族女干部。

　　**第十二条**　各级妇女联合会及其团体会员，可以向国家机关、社会团体、企业事业单位推荐女干部。

　　**第十三条**　对于有关保障妇女权益的批评或者合理建议，有关部门应当听取和采纳；对于有关侵害妇女权益的申诉、控告和检举，有关部门必须查清事实，负责处理，任何组织或者个人不得压制或者打击报复。

第三章　文化教育权益

　　**第十四条**　国家保障妇女享有与男子平等的文化教育权利。

　　**第十五条**　学校和有关部门应当执行国家有关规定，保障妇女在入学、升学、毕业分配、授予学位、派出留学等方面享有与男子平等的权利。

　　**第十六条**　学校应当根据女性青少年的特点，在教育、管理、设施等方面采取措施，保障女性青少年身心健康发展。

　　**第十七条**　父母或者其他监护人必须履行保障适龄女性儿童少年接受义务教育的义务。

　　除因疾病或者其他特殊情况经当地人民政府批准的以外，对不送适龄女性儿童少年入学的父母或者其他监护人，由当地人民政府以批评教育，并采取有效措施，责令送适龄女性儿童少年入学。

　　政府、社会、学校应针对适龄女性儿童少年就学存在的实际困难，采取有效措施，保证适龄女性儿童少年受完当地规定年限的义务教育。

　　**第十八条**　各级人民政府应当依照规定把扫除妇女中的文盲、半文盲工作，纳入扫盲和扫盲后继续教育规划，采取符合妇女特点的组织形式和工作方法，组织、监督有关部门具体实施。

　　**第十九条**　各级人民政府和有关部门应当采取措施，组织妇女接受职业教育和技术培训。

　　**第二十条**　国家机关、社会团体和企业事业单位应当执行国家有关规定，保障妇女从事科学、技术、文学、艺术和其他文化活动，享有与男子平等的权利。

第四章　劳动权益

　　**第二十一条**　国家保障妇女享有与男子平等的劳动权利。

　　**第二十二条**　各单位在录用职工时，除不适合妇女的工种或者岗位外，不得以性别为由拒绝录用妇女或者提高对妇女的录用标准。

　　禁止招收未满十六周岁的女工。

　　**第二十三条**　实行男女同工同酬。

　　在分配住房和享受福利待遇方面男女平等。

　　**第二十四条**　在晋职、晋级、评定专业技术职务等方面，应当坚持男女平等的原则，不得歧视妇女。

　　**第二十五条**　任何单位均应根据妇女的特点，依法保护妇女在工作和劳动时的安全和健康，不得安排不适合妇女从事的工作和劳动。

　　妇女在经期、孕期、产期、哺乳期受特殊保护。

　　**第二十六条**　任何单位不得以结婚、怀孕、产假、哺乳等为由，辞退女职工或者单方解除劳动合同。

　　**第二十七条**　国家发展社会保险、社会救济和医疗卫生事业，为年老、疾病或者丧失劳动能力的妇女获得物质资助创造条件。

第五章　财产权益

　　**第二十八条**　国家保障妇女享有与男子平等的财产权利。

　　**第二十九条**　在婚姻、家庭共有财产关系中，不得侵害妇女依法享有的权益。

　　**第三十条**　农村划分责任田、口粮田等，以及批准宅基地，妇女与男子享有平等权利，不得侵害妇女的合法权益。

　　妇女结婚、离婚后，其责任田、口粮田和宅基地等，应当受到保障。

　　**第三十一条**　妇女享有的与男子平等的财产继承权受法律保护。在同一顺序法定继承人中，不得歧视妇女。

　　丧偶妇女有权处分继承的财产，任何人不得干涉。

　　**第三十二条**　丧偶妇女对公、婆尽了主要赡养义务的，作为公、婆的第一顺序法定继承人，其继承权不受子女代位继承的影响。

第六章　人身权利

　　**第三十三条**　国家保障妇女享有与男子平等的人身权利。

　　**第三十四条**　妇女的人身自由不受侵犯。禁止非法拘禁和以其他非法手段剥夺或者限制妇女的人身自由；禁止非法搜查妇女的身体。

　　**第三十五条**　妇女的生命健康权不受侵犯。禁止溺、弃、残害女婴；禁止歧视、虐待生育女婴的妇女和不育的妇女；禁止用迷信、暴力手段残害妇女；禁止虐待、遗弃老年妇女。

　　**第三十六条**　禁止拐卖、绑架妇女；禁止收买被拐卖、绑架的妇女。

　　人民政府和有关部门必须及时采取措施解救被拐卖、绑架的妇女。被拐卖、绑架的妇女返回原籍的，任何人不得歧视，当地人民政府和有关部门应当做好善后工作。

　　**第三十七条**　禁止卖淫、嫖娼。

　　禁止组织、强迫、引诱、容留、介绍妇女卖淫或者雇用、容留妇女与他人进行猥亵活动。

　　**第三十八条**　妇女的肖像权受法律保护。未经本人同意，不得以营利为目的，通过广告、商标、展览橱窗、书刊、杂志等形式使用妇女肖像。

　　**第三十九条**　妇女的名誉权和人格尊严受法律保护。禁止用侮辱、诽谤、宣扬隐私等方式损害妇女的名誉和人格。

第七章　婚姻家庭权益

　　**第四十条**　国家保障妇女享有与男子平等的婚姻家庭权利。

　　**第四十一条**　国家保护妇女的婚姻自主权。禁止干涉妇女的结婚、离婚自由。

　　**第四十二条**　女方按照计划生育的要求中止妊娠的，在手术后六个月内，男方不得提出离婚；女方提出离婚，或者人民法院认为确有必要受理男方离婚请求的，不在此限。

　　**第四十三条**　妇女对依照法律规定的夫妻共同财产享有与其配偶平等的占有、使用、收益和处分的权利，不受双方收入状况的影响。

　　**第四十四条**　国家保护离婚妇女的房屋所有权。

　　夫妻共有的房屋，离婚时，分割住房由双方协议解决；协议不成的，由人民法院根据双方的具体情况，照顾女方和子女权益的原则判决。夫妻双方另有约定的除外。

　　夫妻共同租用的房屋，离婚时，女方的住房应当按照照顾女方和子女权益的原则协议解决。

　　夫妻居住男方单位的房屋，离婚时，女方无房居住的，男方有条件的应当帮助其解决。

　　**第四十五条**　父母双方对未成年子女享有平等的监护权。

　　父亲死亡、丧失行为能力或者有其他情形不能担任未成年子女的监护人的，母亲的监护权任何人不得干涉。

　　**第四十六条**　离婚时，女方因实施绝育手术或者其他原因丧失生育能力的，处理子女抚养问题，应在有利子女权益的条件下，照顾女方的合理要求。

　　**第四十七条**　妇女有按照国家有关规定生育子女的权利，也有不生育的自由。

　　育龄夫妻双方按照国家有关规定计划生育，有关部门应当提供安全、有效的避孕药具和技术，保障实施节育手术的妇女的健康和安全。

第八章　法律责任

　　**第四十八条**　妇女的合法权益受到侵害时，被侵害人有权要求有关主管部门处理，或者依法向人民法院提起诉讼。

　　妇女的合法权益受到侵害时，被侵害人可以向妇女组织投诉，妇女组织应当要求有关部门或者单位查处，保护被侵害妇女的合法权益。

　　**第四十九条**　违反本法规定侵害妇女合法权益，其他法律、法规已规定处罚的，依照该法律、法规的规定处罚。

　　**第五十条**　有下列侵害妇女合法权益情形之一的，由其所在单位或者上级机关责令改正，并可根据具体情况，对直接责任人员给予行政处分：

　　（一）对有关侵害妇女权益的申诉、控告、检举，推诿、拖延、压制不予查处的；

　　（二）依照法律、法规规定，应当录用而拒绝录用妇女或者对妇女提高录用条件的；

　　（三）在分配住房和晋职、晋级、评定专业技术职务等方面，违反男女平等原则，侵害妇女合法权益的；

　　（四）以结婚、怀孕、产假、哺乳等为由辞退女职工的；

　　（五）划分责任田、口粮田等，以及批准宅基地，违反男女平等原则，侵害妇女合法权益的；

　　（六）在入学、升学、毕业分配、授予学位、派出留学等方面，违反男女平等原则，侵害妇女合法权益的。

　　对侵害妇女权益的行为提出申诉、控告、检举的人进行打击报复的，由其所在单位或者上级机关责令改正或者给予行政处分；国家工作人员进行打击报复构成犯罪的，依照[刑法](https://www.pkulaw.com/chl/29dd76714a5cc235bdfb.html#class=alink)第[一百四十六条](https://www.pkulaw.com/chl/29dd76714a5cc235bdfb.html#%23tiao_146class=alink)的规定追究刑事责任。

　　**第五十一条**　雇用、容留妇女与他人进行猥亵活动的，比照[治安管理处罚条例](https://www.pkulaw.com/chl/5c4f7e992031024bbdfb.html#class=alink)第[十九条](https://www.pkulaw.com/chl/5c4f7e992031024bbdfb.html#%23tiao_19class=alink)的规定处罚，情节严重，构成犯罪的，比照[刑法](https://www.pkulaw.com/chl/29dd76714a5cc235bdfb.html#class=alink)第[一百六十条](https://www.pkulaw.com/chl/29dd76714a5cc235bdfb.html#%23tiao_160class=alink)的规定追究刑事责任。

　　**第五十二条**　侵害妇女的合法权益，造成财产损失或者其它损害的，应当依法赔偿或者承担其他民事责任。

第九章　附则

　　**第五十三条**　国务院有关部门可以根据本法制定有关条例，报国务院批准施行。

　　省、自治区、直辖市人民代表大会常务委员会可以根据本法制定实施办法。

　　民族自治地方的人民代表大会，可以依据本法规定的原则，结合当地民族妇女的具体情况，制定变通的或者补充的规定。自治区的规定，报全国人民代表大会常务委员会备案；自治州、自治县的规定，报省或者自治区人民代表大会常务委员会批准后生效，并报全国人民代表大会常务委员会备案。

　　**第五十四条**　本法自１９９２年１０月１日起施行。

　　附：

法律有关条文
[刑法](https://www.pkulaw.com/chl/29dd76714a5cc235bdfb.html#class=alink)有关条款

　　第一百四十六条　国家工作人员滥用职权、假公济私，对控告人、申诉人、批评人实行报复陷害的，处二年以下有期徒刑或者拘役；情节严重的，处二年以上七年以下有期徒刑。
　　第一百六十条　聚众斗殴，寻衅滋事，侮辱妇女或者进行其他流氓活动，破坏公共秩序，情节恶劣的，处七年以下有期徒刑、拘役或者管制。
　　流氓集团的首要分子，处七年以上有期徒刑。

[治安管理处罚条例](https://www.pkulaw.com/chl/5c4f7e992031024bbdfb.html#class=alink)有关条款

　　第十九条　有下列扰乱公共秩序行为之一，尚不够刑事处罚的，处十五日以下拘留、二百元以下罚款或者警告：
　　……
　　（四）结伙斗殴，寻衅滋事，侮辱妇女或者进行其它流氓活动的；
　　……

© 北大法宝：（[www.pkulaw.com](https://www.pkulaw.com/)) 专业提供法律信息、法学知识和法律软件领域各类解决方案。北大法宝为您提供丰富的参考资料，正式引用法规条文时请与标准文本核对。
欢迎查看所有[产品和服务](https://www.pkulaw.com/helps/2.html)。法宝快讯：[如何快速找到您需要的检索结果？](https://www.pkulaw.com/helps/70.html)[法宝](https://www.pkulaw.com/helps/69.html)V6有何新特色？

扫描二维码阅读原文

**原文链接：**<https://www.pkulaw.com/chl/dff2eefbddf155b3bdfb.html>
